# Diagnostic accuracy of a noninvasive hepatic ultrasound score for non-alcoholic fatty liver disease (NAFLD) in the Brazilian Longitudinal Study of Adult Health (ELSA-Brasil): Acurácia diagnóstica de um escore de ultrassonografia hepática não invasivo para doença hepática gordurosa não alcoólica (DHGNA) no Estudo Longitudinal de Saúde do Adulto (ELSA-Brasil)

**DOI:** 10.1590/1516-3180.2014.9150812

**Published:** 2014-11-28

**Authors:** Alessandra Carvalho Goulart, Ilka Regina Souza de Oliveira, Airlane Pereira Alencar, Maira Solange Camara dos Santos, Itamar Souza Santos, Brenda Margatho Ramos Martines, Danilo Peron Meireles, João Augusto dos Santos Martines, Giovanni Misciagna, Isabela Martins Benseñor, Paulo Andrade Lotufo

**Affiliations:** I MD, PhD. Clinical Epidemiologist and Researcher, Center for Clinical and Epidemiological Research, Hospital Universitário, Universidade de São Paulo (HU-USP), São Paulo, Brazil.; II MD, PhD. Professor, Radiology Department, Faculdade de Medicina da Universidade de São Paulo (FMUSP), São Paulo, Brazil.; III MD, PhD. Professor of Statistics and Mathematics, Institute of Mathematics and Statistics, Universidade de São Paulo (USP), São Paulo, Brazil.; IV MD. Researcher, Center for Clinical and Epidemiological Research, Hospital Universitário, Universidade de São Paulo (HU-USP), São Paulo, Brazil.; V MD, PhD. Professor, Center for Clinical and Epidemiological Research, Hospital Universitário, Universidade de São Paulo (HU-USP), São Paulo, Brazil. São Paulo, Brazil.; VI MD. Attending Physician, Radiology Department, Faculdade de Medicina da Universidade de São Paulo (FMUSP), São Paulo, Brazil.; VII Radiology Technician, Center for Clinical and Epidemiological Research, Hospital Universitário, Universidade de São Paulo (HU-USP), São Paulo, Brazil.; VIII MD. Attending Physician, Radiology Department, Faculdade de Medicina da Universidade de São Paulo (FMUSP), São Paulo, Brazil.; IX MD. Researcher Ethics Committee, University Hospital, University of Bari, Italy.; X MD, PhD. Professor of Department of Internal Medicine and Director of Center for Clinical and Epidemiological Research, Hospital Universitário, Universidade de São Paulo (HU-USP), São Paulo, Brazil.; XI MD, PhD. Professor of Department of Internal Medicine and Head of the Center for Clinical and Epidemiological Research, Hospital Universitário, Universidade de São Paulo (HU-USP), São Paulo, Brazil.

**Keywords:** Liver disease, Epidemiology, Diagnostic techniques and procedures, Ultrasonography, Tomography, x-ray computed

## Abstract

**CONTEXT AND OBJECTIVE:**

Noninvasive strategies for evaluating non-alcoholic fatty liver disease (NAFLD) have been investigated over the last few decades. Our aim was to evaluate the diagnostic accuracy of a new hepatic ultrasound score for NAFLD in the ELSA-Brasil study.

**DESIGN AND SETTINGS:**

Diagnostic accuracy study conducted in the ELSA center, in the hospital of a public university.

**METHODS:**

Among the 15,105 participants of the ELSA study who were evaluated for NAFLD, 195 individuals were included in this sub-study. Hepatic ultrasound was performed (deep beam attenuation, hepatorenal index and anteroposterior diameter of the right hepatic lobe) and compared with the hepatic steatosis findings from 64-channel high-resolution computed tomography (CT). We also evaluated two clinical indices relating to NAFLD: the fatty liver index (FLI) and the hepatic steatosis index (HSI).

**RESULTS:**

Among the 195 participants, the NAFLD frequency was 34.4%. High body mass index, high waist circumference, diabetes and hypertriglyceridemia were associated with high hepatic attenuation and large anteroposterior diameter of the right hepatic lobe, but not with the hepatorenal index. The hepatic ultrasound score, based on hepatic attenuation and the anteroposterior diameter of the right hepatic lobe, presented the best performance for NAFLD screening at the cutoff point ≥ 1 point; sensitivity: 85.1%; specificity: 73.4%; accuracy: 79.3%; and area under the curve (AUC 0.85; 95% confidence interval, CI: 0.78-0.91)]. FLI and HSI presented lower performance (AUC 0.76; 95% CI: 0.69-0.83) than CT.

**CONCLUSION:**

The hepatic ultrasound score based on hepatic attenuation and the anteroposterior diameter of the right hepatic lobe has good reproducibility and accuracy for NAFLD screening.

## RESUMO

**CONTEXTO E OBJETIVO:**

Estratégias não invasivas para avaliar doença hepática gordurosa não alcoólica (DHGNA) têm sido investigadas nas últimas décadas. Nosso objetivo foi avaliar a acurácia diagnóstica de um novo escore de ultrassonografia hepática para DHGNA no estudo ELSA-Brasil.

**TIPO DE ESTUDO E LOCAL:**

Estudo de acurácia diagnóstica realizado no centro ELSA, no hospital de uma universidade pública.

**MÉTODOS:**

Dos 15.105 participantes do estudo ELSA avaliados para DHGNA, 195 indivíduos foram incluídos neste estudo. Foi realizada ultrassonografia hepática (atenuação do feixe de profundidade, índice hepatorrenal e diâmetro anteroposterior do lobo direito do fígado) que foi comparada aos achados de esteatose hepática da tomografia computadorizada (TC) de alta resolução de 64 canais. Avaliamos também dois índices clínicos relacionados à DHGNA: o índice de gordura hepática (FLI) e o índice de esteatose hepática (HSI).

**RESULTADOS:**

Entre os 195 participantes, a frequência de DHGNA foi de 34,4%. Altos índices de massa corpórea, circunferência de cintura, diabetes e hipertrigliceridemia foram associados a aumento da atenuação hepática e do diâmetro anteroposterior do lobo hepático direito, mas não ao índice hepato-renal. A pontuação da ultrassonografia hepática, que incluiu atenuação hepática e diâmetro anteroposterior do lobo direito do fígado, apresentou o melhor desempenho para o rastreio DHGNA sob o ponto de corte ≥ 1 ponto (sensibilidade: 85.1%; especificidade: 73.4%; acurácia: 79.3%), e área sob a curva (AUC, 0,85, IC 95%: 0,78-0,91). FLI e HSI apresentaram menor desempenho (AUC 0,76; IC 95%: 0,69-0,83) comparados à TC.

**CONCLUSÃO:**

O escore de ultrassonografia hepática, que incluiu atenuação hepática e o diâmetro anteroposterior do lobo direito do fígado, possui boa reprodutibilidade e acurácia para o rastreio de DHGNA.

## INTRODUCTION

Nonalcoholic fatty liver disease (NAFLD) comprises a spectrum of conditions ranging from hepatic steatosis to fibrosis^1^ and has also been associated with endothelial dysfunction,^2^ metabolic syndrome,^3^ insulin resistance,^4^ type 2 diabetes,^5^ dyslipidemia,^5^ and independent cardiovascular events.^6^^,^^7^^,^^8^ Liver biopsy is considered to be the gold standard for diagnosing and staging NAFLD.^1^ However, its use is limited by cost, sampling error and adverse events. Furthermore, liver biopsy is not feasible for population-based studies and cohort studies.

Interest in noninvasive imaging strategies for NAFLD screening, including conventional B-mode ultrasound, has increased significantly over the last decade.^9^^,^^10^^,^^11^^,^^12^^,^^13^^,^^14^ Although computed tomography (CT) and magnetic resonance imaging (MRI) are superior to conventional B-mode ultrasound for detecting fatty liver infiltration, their costs and adverse effects limit their usefulness in clinical practice, field surveys, epidemiological studies and clinical trials.^11^^,^^12^^,^^13^^,^^14^^,^^15^^,^^16^^,^^17^^,^^18^ The positive aspects of B-mode ultrasound, such as being routinely available and completely safe, make it a valuable tool in clinical settings and large population studies.^13^^,^^14^^,^^15^^,^^16^^,^^17^^,^^18^^,^^19^^,^^20^^,^^21^^,^^22^^,^^23^^,^^24^

The most common B-mode ultrasound criteria reported for diffuse hepatic fatty infiltration are as follows: (1) parenchymal brightness; (2) deep-beam attenuation; (3) vascular blurring (loss of echoes from the walls of the portal veins); and (4) increasing discrepancy of echogenicity between the liver and kidney parenchyma (hepatorenal index).^1^^,^^10^^,^^15^^,^^19^^,^^20^^,^^21^^,^^22^ Among these criteria, deep-beam attenuation has presented better performance for diagnosing fatty liver disease.^25^^,^^26^^,^^27^^,^^28^^,^^29^^,^^30^^,^^31^

However, conventional B-mode ultrasonography presents limitations, including subjective evaluation and operator dependency. Therefore, inclusion of a new biometric parameter, i.e. measurement of the anteroposterior diameter of the right hepatic lobe, was proposed in a Brazilian cohort of 15,105 civil servants aged 35 to 74 years in a study that was designed to evaluate the risk factors for diabetes and cardiovascular diseases: the Longitudinal Study for Adult Health (in Portuguese, “Estudo Longitudinal de Saúde do Adulto”, known as ELSA-Brasil).^32^^,^^33^^,^^34^^,^^35^ The rationale behind using the anteroposterior diameter of the right hepatic lobe was the significant association with hepatic steatosis, confirmed by liver biopsy, that had previously been described.^36^

## OBJECTIVE

Thus, the purpose of this study was to evaluate the diagnostic accuracy of a new hepatic ultrasound score for assessing NAFLD based on B-mode ultrasound deep-beam attenuation, the anteroposterior diameter of the right hepatic lobe and the hepatorenal index, compared with 64-channel multidetector CT findings (64-MDCT) in an ELSA-Brasil subsample.

The performance of 64-MDCT versus two clinical indices relating to NAFLD was also evaluated: the fatty liver index (FLI)^25^ and the hepatic steatosis index (HSI).^37^

## METHODS

### Study design

This was a diagnostic accuracy study conducted on a subsample from the ELSA-Brasil study.

All active or retired employees of both sexes aged 35 to 74 years, at six institutions across the country, in Belo Horizonte, Porto Alegre, Rio de Janeiro, São Paulo, Salvador and Vitoria, were eligible for the study. In 2008, these totaled 52,137 potential participants. We chose civil servants as the source of the study population in order to minimize losses from the follow-up relating to geographical mobility. The exclusion criteria were severe cognitive or communication impairment, intention to quit work at the institution in the near future for reasons unrelated to retirement and, if retired, residence outside the corresponding metropolitan area. Participation by women with current or recent pregnancy was rescheduled so that the first interview could take place four months after delivery. In total, 15,105 individuals were enrolled as participants in the ELSA-Brasil study at the six different sites between August 2008 and December 2010. All of the participants underwent a baseline conventional B-mode liver ultrasound for evaluation of NAFLD. At the same time, biomarker data such as serum lipid and hepatic enzyme levels associated with NAFLD were collected.^34^ Full descriptions of the ELSA-Brasil study have been published elsewhere.^32^^,^^33^^,^^34^^,^^35^

### Sampling and settings

Our reference population was recruited at the ELSA-São Paulo site (n = 5,061 participants) and underwent liver ultrasound scanning one year before this sub-study, during the baseline visit. The ELSA-São Paulo site is located at the university hospital of the University of São Paulo (HU-USP), in the western area of the city of São Paulo, the largest metropolitan area in South America. HU-USP was the only facility in this area of study, which includes 420,000 inhabitants.

For sampling purposes, participants who presented the following baseline characteristics were not included in this sub-study: (1) self-reported alcohol intake above 40 g per day for men and 20 g per day for women; (2) self-reported liver disease; (3) positive serological tests for the hepatitis B virus and C virus; (4) values ≥ four times the upper limit of normality for serum alanine aminotransferase (ALT), aspartate aminotransferase (AST) and gamma-glutamyl transferase (GGT); and (5) baseline liver ultrasound scans showing heterogeneous parenchymal texture, focal lesions or ascites.

By taking the statistical power to be 80% and the prevalence of NAFLD to be 30%, based on the general population,^1^ the sample size was estimated as 176 individuals.

In total, 195 participants were invited to participate in this sub-study and none were lost during the follow-up. On the same day on which they returned to HU-USP to participate in this sub-study, they underwent new NAFLD biomarker testing, new liver ultrasound scanning and, lastly, an abdominal 64-MDCT scan to determine any presence of liver steatosis.

Written informed consent was obtained from all participants who agreed to participate in this study, and each subject received a copy of the consent form. The Internal Review Board of the University of São Paulo approved both the main study and this sub-study.

Furthermore, the recommendations of the Standards for Reporting of Diagnostic Accuracy (STARD) for publication of accuracy studies were followed.

### NAFLD biomarkers

For all of these 195 ELSA-Brasil participants, the fatty liver index (FLI) and the hepatic steatosis index (HIS) were applied as surrogate outcomes for hepatic steatosis, as described below:

1. Fatty liver index (FLI): Bedogni et al.^25^ validated the FLI against the ultrasound findings of hepatic steatosis in a Mediterranean population from the Dionysios Nutrition & Liver Study. The FLI was calculated according to the following algorithm described in the original study: FLI = (e 0.953*log_e_ (triglycerides) + 0.139*BMI (body mass index) + 0.718*log_e_ (GGT) + 0.053*waist circumference - 15.745)/(1 + e 0.953*log_e_ (triglycerides) + 0.139*BMI + 0.718*log_e_ (GGT) + 0.053*waist circumference - 15.745) * 100. FLI ranges from 0 to 100 and suggests that fat infiltration is present at a cutoff point greater than or equal to 60 points, with sensitivity and specificity of 61% and 86%, respectively.
2. Hepatic steatosis index (HSI): this index was validated in a cross-sectional study conducted in a health check-up on the Korean population.^37^ In this study, multivariate analysis indicated the main factors associated with hepatic steatosis, compared with US findings, and the following equation was derived: HSI = 8 x (ALT/AST ratio) + BMI (+2, if female; +2, if diabetes mellitus). The HSI discarded NAFLD cases at a cutoff point lower than 30, with a sensitivity of 93.1%, and confirmed the diagnosis at a cutoff greater than 36, with a specificity of 92.4%.

Anthropometric parameters, such as weight and height to calculate BMI [weight (kg)/height (m)^2^] and waist circumference (cm) were measured using standard equipment and techniques.^35^ Serum biochemistry parameters were evaluated using the enzymatic method (International Federation of Clinical Chemistry, IFCC; modified) performed on an Advia Siemens system for ALT and AST and the colorimetric kinetic method (Szasz) performed on the Advia Siemens system for GGT.^32^ Liver tests were considered abnormal if they were at least one and half points above the upper limit of the normal reference values, as follows: ALT normal range: 10 to 35 IU/l for men and 10 to 31 IU/l for women; AST normal range: 9 to 43 IU/l for men and 9 to 36 IU/l for women; and GGT normal range: up to 30 IU/l for men and up to 24 IU/l for women.^34^ Fasting glucose (hexoquinase) and serum triglycerides (glycerol phosphate peroxidase) were also analyzed. Diabetes mellitus was defined as fasting glucose > 126 mg/dl or treatment with an anti-diabetic medication.^37^

### Hepatic ultrasound protocol

Liver ultrasound examinations on the participants were performed by board-certified radiologists or by radiology technicians, after adequate training, using the same models of equipment: a high-resolution B-mode scanner (SSA-790A, Aplio XG, Toshiba Medical System, Tokyo, Japan) and a convex array transducer (model PVT-375BT), with a central frequency of 3.5 MHz, and a fundamental frequency of 1.9-5.0 MHz. After the acquisition process, the B-mode hepatic ultrasound images were read by board-certified radiologists at the ELSA-São Paulo site, which was established as the ELSA-Brasil ultrasound reading center. The quality control protocol was verified by a senior ultrasound radiologist by crosschecking the data. The liver ultrasound scanning protocol was set up in accordance with the following criteria:

1. Hepatic attenuation of the ultrasound beam: A standard B-mode ultrasound evaluation was made using a four-point visual grading system based on the degree of diaphragm viewing posterior to the right hepatic lobe. The hepatic attenuation was classified as normal (complete viewing of the diaphragm) or abnormal as follows: mild (partial, i.e. > 50% viewing of the diaphragm); moderate (partial, i.e. < 50% viewing of the diaphragm); or severe (no viewing of the diaphragm).
2. Anteroposterior diameter of the right hepatic lobe: This parameter was measured on the images in the axial plane by scanning between the intercostal spaces. The ultrasound images would present, at best, four anatomical landmarks: the anterior and posterior hepatic surfaces, the gallbladder and the portal vein.
3. Hepatorenal index: This was the ratio between the brightness level in the hepatic parenchyma and that in the renal parenchyma, based on qualitative assessment of ultrasound images. Fatty liver disease was diagnosed with a hepatorenal index greater than or equal to 1.5.^19^^,^^20^

The quality control for the ultrasound images obtained from the anteroposterior diameter measurement on the right hepatic lobe was based on the number of required anatomical landmarks in the axial ultrasound plane of the right hepatic lobe, as follows: (1) unacceptable (no anatomical landmark); (2) poor (one anatomical landmark); (3) acceptable (two anatomical landmarks: anterior and posterior hepatic surfaces); (4) very good (three anatomical landmarks: anterior and posterior hepatic surfaces, gallbladder or portal vein); and (5) excellent (four anatomical landmarks: anterior and posterior hepatic surfaces, gallbladder and portal vein).

### Hepatic CT examination protocol

Imaging was performed using a Brilliance 64-MDCT scanner (Phillips, Bets, Netherlands). An enhanced 64-MDCT scan, which is a highly sensitive method for estimating hepatic fat, was performed to evaluate hepatic attenuation in Hounsfield units (HU).^38^^,^^39^^,^^40^^,^^41^^,^^42^^,^^43^^,^^44^ The indices applied in our inter-method comparison of fatty liver disease diagnoses were as follows:

1. Hepatic attenuation index: Based on unenhanced CT, the attenuation values measured in the normal liver (range 50-65 HU) are typically higher than those measured in the spleen, which has been attributed to the presence of glycogen in the liver. Fatty infiltration was diagnosed with hepatic attenuation < 48 HU.^40^^,^^41^
2. Hepatic-splenic difference: The mean densities of the liver and spleen were calculated from the regions of interest. The mean liver-to-spleen attenuation difference for each patient was determined by subtracting the mean splenic attenuation from the mean liver attenuation. Based on previous studies, a hepatic-splenic attenuation difference of more than 5 HU is an accurate predictor of the absence of significant macrovesicular steatosis (0%-5%), and a range from -10 to 5 HU is suggestive of mild to moderate steatosis (6%-30%); in our study, 5 HU was the cutoff point used for hepatic steatosis.^43^

All examiners who performed ultrasound and CT exams were kept blinded with regard to the NAFLD diagnosis.

### Statistics

The qqplot graphic and the Kolmogorov-Smirnov test were applied as normality tests to evaluate the distribution of continuous variables in our sample.

Additionally, the χ^2^ test for categorical variables and the Kruskal-Wallis or Mann-Whitney test for continuous variables were used to evaluate associations among the baseline characteristics (including sociodemographic and anthropometric data), in accordance with the NAFLD ultrasound criteria, independently:

1. Hepatic deep-beam attenuation to three degrees (1. normal; 2. mild; 3. moderate or severe);
2. Hepatorenal index (< 1.5 versus ≥ 1.5, without and with hepatic steatosis, respectively);
3. Anteroposterior diameter cutoff points for the right hepatic lobe were estimated according to the hepatic steatosis detected by means of 64-MDCT (based on the hepatic attenuation index and hepatic-splenic difference), as follows: ≥ 119.5 mm (men) or ≥ 102.5 mm (women).

Sensitivity, specificity, accuracy, receiver operating characteristic (ROC) curve, plus the area under the curve (AUC), were performed to measure the discriminatory power of our ultrasound scoring system for detecting hepatic steatosis compared with 64-MDCT as gold standard. The same indexes were also determined in order to evaluate the accuracy of the FLI and the HIS, in comparison with 64-MDCT, on the same subsample of ELSA-Brasil. For these indexes, the cutoffs proposed by the original articles were applied, as follows:^25^^,^^37^ FLI: < 30 (rule out) versus ≥ 60 (rule in); HSI: < 30 (rule out) versus > 36 (rule in).

All tests were two-tailed, and P-values < 0.05 were considered significant. Statistical analyses were performed using the statistical package for the social sciences (SPSS) software, version 16.0, and the free R software.

## RESULTS

The frequency of hepatic steatosis according to the 64-MDCT criteria among the 195 ELSA-Brasil participants was 34.4%, regardless of gender. Baseline characteristics, including sociodemographic factors, preclinical comorbidities and biomarkers, are shown in Table 1.

**Table 1.**
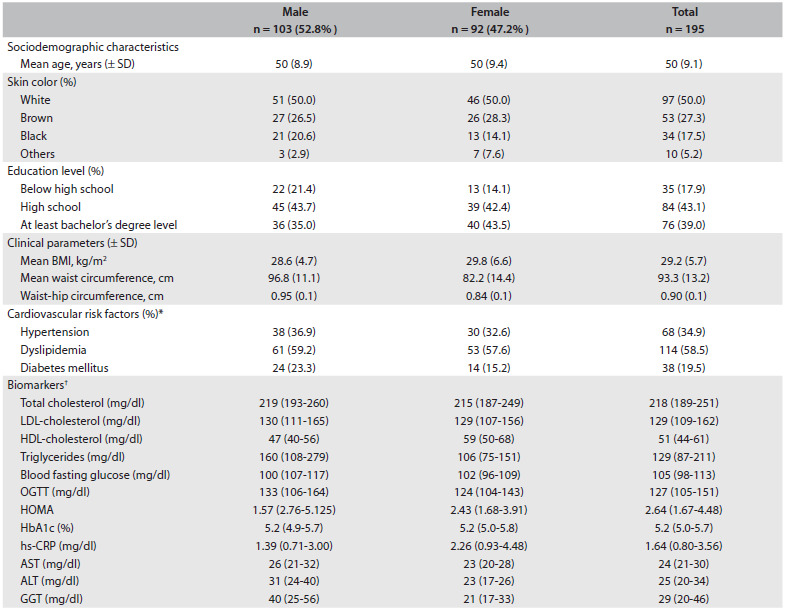
Baseline characteristics of 195 participants from the ELSA study according to gender

*Hypertension diagnosis was based on the Seventh Report of the Joint National Committee on Prevention. Detection, Evaluation and Treatment of High Blood Pressure (Hypertension 2003); ^†^Continuous variables are expressed as the median (interquartile interval); Dyslipidemia diagnosis was based on the Third Report of the National Cholesterol Education Program (NCEP) Expert Panel on Detection, Evaluation and Treatment of High Blood Cholesterol in Adults (Adult Treatment Panel III) final report (Circulation 2002) and diabetes mellitus diagnoses are based on Diagnosis and Classification of Diabetes Mellitus, American Diabetes Association (Diabetes Care 2010). SD = standard deviation; BMI = body mass index; OGTT = oral glucose tolerance test; HbA1c = glycosylated hemoglobin; hs-CRP = high-sensitivity C-reactive protein; AST = aspartate aminotransferase; ALT = alanine aminotransferase; GGT = gamma-glutamyl transferase; ELSA = Estudo Longitudinal de Saúde do Adulto - Brasil. Some proportions might not add up to 100% due to rounding.

**Figure 1.**
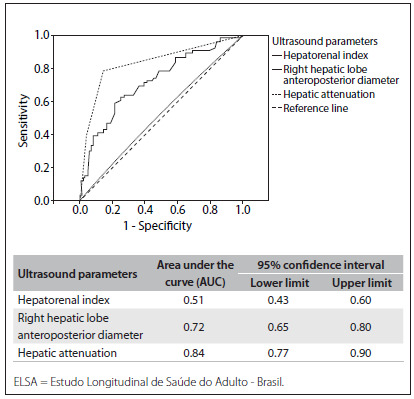
Receiver operator characteristic curve (ROC) for ultrasound parameters for assessing non-alcoholic fatty liver disease (NAFLD), compared with hepatic steatosis diagnosis performed using computed tomography (CT), on 195 ELSA-Brasil participants.

Figure 1 shows the ROC curves of the three ultrasound criteria, using 64-MDCT as the gold standard for determining liver steatosis. The hepatorenal index was found to have worse performance (AUC: 0.51; 95% confidence interval. CI: 0.43-0.60), in comparison with the anteroposterior diameter of the right hepatic lobe (AUC: 0.72; 95% CI: 0.65-0.80) and the hepatic attenuation of the ultrasound beam criteria (AUC: 0.84; 95% CI: 0.77-0.90).

Table 2 shows the associations of the characteristics of the participants in this study, according to increased hepatic deep-beam attenuation (divided into normal, mild and moderate-severe), hepatorenal index (no steatosis < 1.5 versus steatosis ≥ 1.5*)* and anteroposterior diameter of the right hepatic lobe (cutoff points of ≥ 119.5 mm for men and ≥ 102.5 mm for women). It should be noted that these best cutoff values for the anteroposterior diameter of the right hepatic lobe were previously evaluated by means of ROC analysis, among the same 195 participants from the present sub-study, and they differentiated individuals with hepatic steatosis from those without hepatic steatosis, with good sensitivity (59% and 65%) and specificity (84.8% and 78.1%) for men and women, respectively.

**Table 2.**
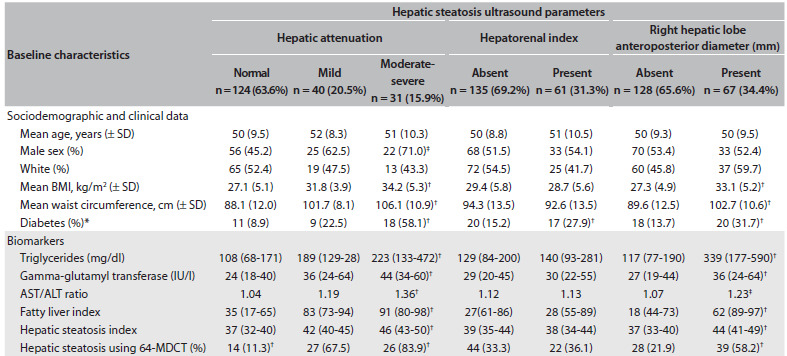
Characteristics in 195 participants from the ELSA study according to sonogram parameters to identify hepatic steatosis

Biomarkers and hepatic steatosis indexes are expressed as median (interquartile interval). Hepatic steatosis index (HIS) = [8 X (ALT/AST ratio) +BMI (+2. if female; +2 if diabetes mellitus)]. Fatty liver index (FLI) = (e 0.953*log_e_ (triglycerides) + 0.139*BMI + 0.718*log_e_ (GGT) + 0.053*waist circumference - 15.745)/(1 + e 0.953*log_e_ (triglycerides) + 0.139*BMI + 0.718*log_e_ (GGT) + 0.053*waist circumference - 15.745) * 100. 64-MDCT: 64-channel multidetector computed tomography. Anteroposterior diameter of the right hepatic lobe (mm) ≥ 119.5 (men) or ≥ 102.5 (women) was defined as suggestive of hepatic steatosis and < 119.5 (men) or < 102.5 (women) as absence of this condition. ELSA = Estudo Longitudinal de Saúde do Adulto - Brasil. *P-values were obtained from the chi-square test for categorical variables and from the Kruskal-Wallis or Mann-Whitney test for continuous variables; ^†^P-value ≤ 0.001, ^‡^P-value ≤ 0.05.

Hepatic attenuation and the anteroposterior diameter of the right hepatic lobe, evaluated independently, were significantly associated (P < 0.001) with steatosis seen by means of 64-MDCT. In contrast, no significant association was obtained when the hepatorenal index was evaluated by itself.

High BMI, high waist circumference and diagnoses of diabetes and hypertriglyceridemia were associated with increased hepatic deep-beam attenuation and increased anteroposterior diameter of the right hepatic lobe seen by means of B-mode ultrasound. However, only diabetes was positively associated with a hepatorenal index result suggestive of fatty liver.

**Figure 2.**
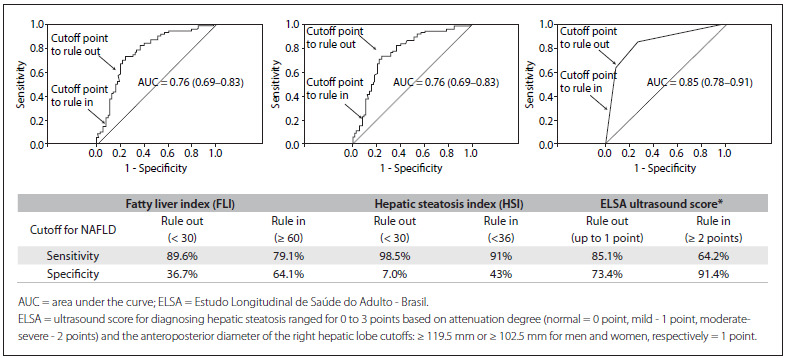
Receiver operator characteristic curve (ROC) for fatty liver index, hepatic steatosis index and ELSA ultrasound score for assessing non-alcoholic fatty liver disease (NAFLD), compared with hepatic steatosis diagnosis made using computed tomography (CT), on 195 ELSA-Brasil participants.

Considering the low accuracy of the hepatorenal index for identifying individuals with hepatic steatosis, a hepatic ultrasound scoring system based exclusively on the following criteria was also explored: hepatic attenuation degree (mild: +1 point and moderate-severe: +2 points) and a cutoff for the anteroposterior diameter of the right hepatic lobe ≥ 119.5 mm (men) or ≥ 102.5 mm (women) (+ 1 point), with a maximum score of 3 points. The hepatic ultrasound score based on hepatic attenuation and the anteroposterior diameter of the right hepatic lobe presented the best performance at a cutoff point = 1 point (sensitivity: 85.1%; specificity: 73.4%; and accuracy: 79.3%) for NAFLD screening, compared with 64-MDCT, with the highest area under the curve (AUC 0.85; 95% CI: 0.78-0.91). The presence of two or more points in our score increased the specificity (91.4%); however, lower sensitivity (64.2%) for screening hepatic steatosis was observed (Figure 2).

The FLI and HSI presented the same lower performance (AUC: 0.76; 95% CI: 0.69-0.83), compared with the hepatic ultrasound score proposed here.

## DISCUSSION

In this subsample of the Brazilian Longitudinal Study of Adult Health (ELSA-Brasil), a positive association was found between hepatic ultrasound score and fatty liver disease. For the hepatic ultrasound scoring system to screen for NAFLD, the combination of increased hepatic deep-beam attenuation and increased anteroposterior diameter of the right hepatic lobe (one or the other being suggestive of hepatic steatosis) was able to separate individuals with this clinical condition from those without it, with 85.1% sensitivity and 73.4% specificity. Additionally, in our study, increased anteroposterior diameter of the right hepatic lobe and hepatic deep-beam attenuation on ultrasound were positively associated with obesity, diabetes, hypertriglyceridemia and greater waist circumference.

Clinical indices such as the FLI and HIS tested on our sample exhibited lower performance for detecting NAFLD than that of our hepatic ultrasound score.

The main feature of the hepatic ultrasound score applied in this study for screening for NAFLD was the inclusion of two B-mode ultrasound criteria: one of them qualitative (hepatic deep-beam attenuation) and the other quantitative (anteroposterior diameter of the right hepatic lobe). These parameters were chosen after taking into account the better performance of hepatic deep-beam attenuation among other sonographic criteria for diagnosing fatty liver disease and the positive association between the anteroposterior diameter of the right hepatic lobe and hepatic steatosis described previously.^26^^,^^27^^,^^28^^,^^29^^,^^30^^,^^36^

Moreover, these two B-mode ultrasound criteria were included in our ultrasound score to meet the requirement of selecting the most reliable, feasible and practical B-mode ultrasound parameters that could be evaluated on standardized images obtained at all site locations included in this longitudinal population study.

According to a meta-analysis conducted by Hernaez et al.^15^ which included 49 studies that provided cross-tabulations of ultrasonography versus histology or standard imaging techniques, the overall sensitivity, specificity, positive likelihood ratio and negative likelihood ratio of ultrasound for detecting moderate-severe fatty liver were 84.8% (95% CI: 79.5-88.9), 93.6% (87.2-97.0), 13.3 (6.4-27.6) and 0.16 (0.12-0.22), respectively. The AUC was 0.93 (0.91-0.95). In this meta-analysis, the diagnosis of hepatic steatosis based on B-mode ultrasound included evaluations of liver parenchymal brightness, qualitative hepatorenal index assessment, hepatic deep beam attenuation, bright vessel walls and gallbladder wall definition. In comparison with this meta-analysis, the ELSA-Brasil hepatic ultrasound score showed similar results for sensitivity and inferior results for specificity, which may be explained by the fact that only two ultrasound parameters were included in our analysis. In our study, the qualitative hepatorenal index assessment did not show any relationship with the clinical parameters associated with NAFLD, and it was not an accurate method for investigating hepatic steatosis, compared with 64-MDCT. However, it is important to note that in our study, the ultrasound evaluations on hepatic and renal parenchyma brightness did not use semi-quantitative methods (such as histogram echo intensity). According to previous studies, the hepatorenal index is an accurate method for quantifying fat infiltration in the liver, but mainly in populations without kidney diseases.^22^ In fact, comparison between the kidney and liver parenchyma for detecting fat infiltration using ultrasound in a population-based study would be compromised because the prevalence of fatty kidney is not negligible and may be associated with cardiovascular risk factors.^45^

In this sub-study on ELSA-Brasil, relatively high frequency of NAFLD was found: greater than 30% as assessed using 64-MDCT. This is concordant with other studies, such as a population-based study conducted in the Mediterranean area, which reported NAFLD prevalence measured using ultrasound of 36.8% for men and 25.7% for women.^14^ In the multiethnic Dallas Heart Study, a total prevalence of NAFLD of 31%, assessed using MR spectroscopy, was reported.^46^

The high prevalence of NAFLD and other cardiometabolic risk factors found in our subsample and elsewhere suggest that the hepatic ultrasound score, based on B-mode ultrasound deep-beam attenuation and the anteroposterior diameter of the right hepatic lobe, may have clinical significance in the context of NAFLD studies, given its good sensitivity, specificity and applicability for population screening purposes.

The FLI and HSI are clinical indices for NAFLD screening that have been described in other samples.^25^^,^^37^ In our study, they demonstrated lower performance than our hepatic ultrasound score. Moreover, using one examination as a strategy for screening for NAFLD has the advantage of being more cost-efficient while maintaining sensitivity and specificity and does not require evaluation of additional biomarkers, as suggested by other clinical indices.^25^^,^^37^

Our study has limitations, such as the small sample size, which did not have sufficient power to perform ROC analyses across all degrees of hepatic steatosis (mild, moderate and severe). In addition, in this subsample, 64-MDCT was used to assess cross-tabulations of ultrasonography versus a standard imaging technique, because hepatic biopsy would not be feasible due to ethical constraints.

Finally, considering the statistical data obtained, it is probable that the hepatic ultrasound score, based on B-mode ultrasound deep-beam attenuation and the anteroposterior diameter of the right hepatic lobe, will be applicable in the context of NAFLD studies, as previously reported in the literature with regard to the importance of assessing hepatic steatosis using noninvasive methods.^47^^,^^48^ Meanwhile, following these preliminary results, we intend to apply this hepatic scoring system to the entire ELSA-Brasil cohort, which is composed of 15,105 participants.

## CONCLUSIONS

The hepatic ultrasound score based on hepatic attenuation and the anteroposterior diameter of the right hepatic lobe is reliable and presented good accuracy for screening for NAFLD.
